# Secondhand smoke risk perception and smoke-free rules in homes: a cross-sectional study in Barcelona (Spain)

**DOI:** 10.1136/bmjopen-2016-014207

**Published:** 2017-01-19

**Authors:** Cristina Lidón-Moyano, Jose M Martínez-Sánchez, Marcela Fu, Montse Ballbè, Juan Carlos Martín-Sánchez, Cristina Martínez, Esteve Fernández

**Affiliations:** 1 Biostatistics Unit, Department of Basic Sciences, Universitat Internacional de Catalunya, Sant Cugat del Vallès, Spain; 2 Tobacco Control Unit, Cancer Prevention and Control Program, Institut Català d'Oncologia, L'Hospitalet de Llobregat, Barcelona, Spain; 3 Cancer Prevention and Control Group, Institut d'Investigació Biomèdica de Bellvitge—IDIBELL, L'Hospitalet de Llobregat, Barcelona, Spain; 4 Department of Clinical Sciences, School of Medicine, Universitat de Barcelona, Barcelona, Spain; 5 Addictions Unit, Institute of Neurosciences, Hospital Clínic de Barcelona, Barcelona, Spain; 6 Medicine and Health Sciences School, Universitat Internacional de Catalunya, Sant Cugat del Vallès, Barcelona, Spain

**Keywords:** Smoke-free legislation, Smoke-free home, Smoking, Secondhand smoke

## Abstract

**Objective:**

To describe the voluntary adoption of smoke-free homes in Spain among general population and to identify variables associated with its voluntary adoption.

**Methods:**

Cross-sectional study of a representative sample (n=731) of the adult population (>26 years) of Barcelona, Spain, in 2013–2014. We defined smoking rules inside the households as complete indoor rules (when smoking was not allowed inside the house), and partial or absent indoor rules (when smoking was allowed in some designated places inside the house or when smoking was allowed everywhere) and described them according to the perceived risk of the secondhand smoke (SHS) exposure. We calculated the prevalence and prevalence ratios (PR) according to sociodemographic variables.

**Results:**

57.4% of households had complete indoor smoke-free rules. The prevalence of households with complete indoor rules was higher among women (PRa: 1.15; 95% CI 1.00 to 1.33), married (PRa: 1.18; 95% CI 1.01 to 1.38), never-smokers (PRa: 2.68; 95% CI 2.06 to 3.50) and in households where a minor lived (PRa: 1.40; 95% CI: 1.20–1.65). Believe that breathing tobacco smoke from smokers is dangerous for non-smokers (PRa: 1.77; 95% CI: 1.06–2.97) is associated with the voluntary adoption of complete indoor smoke-free home.

**Conclusions:**

Risk perceptions of SHS exposure were associated with the voluntary adoption of indoor smoke-free homes.

**Strengths and limitations of this study**

- There is scarce evidence about the relationship between voluntary adoption of smoke-free homes and the risk perception of secondhand smoke exposure.
- One strength of our study is the use of a face-to-face questionnaire with trained interviewers, we potentially increase the internal validity of our results when compared with internet and self-administered surveys because avoid misinterpretation of the questions.
- The main limitation of this study is the potential bias of participation due to the attrition of the cohort of participants. However, all analyses used weighted data to generate representative estimates of the city of Barcelona.
- The study was conducted only in the city of Barcelona, and generalisation of the results to the rest of Spain should be cautious.
- Another potential limitation is the cross-sectional nature of the data, which allows one to establish associations but not to infer causality.

## Introduction

The health consequences of secondhand smoke (SHS) exposure on non-smoker's are well known.^1^ Moreover, passive exposure could be due to different settings such as workplaces, public places (bars, restaurants, etc), public transport or private places. For this reason, since the introduction of the WHO Framework Convention on Tobacco Control (WHO FCTC), many countries have implemented smoke-free policies in public and workplaces to reduce the impact of SHS exposure in non-smoker's health; consequently, there has been a reduction in SHS exposure after their implementation in workplaces and public places.^2^ However, private settings (mainly cars and homes) are never or rarely included in tobacco control policies. Nevertheless, the household is usually the main source of exposure to SHS in children.^3^ ^4^ In addition, children are especially vulnerable to SHS exposure because they breathe more rapidly and inhale more pollutants per pound of body weight than adults.^5^ In addition, SHS exposure is a risk for infant death syndrome, acute respiratory infections, ear problems and mental disorders in children.^6^ ^7^ Accordingly, the harmful effects of passive exposure in private venues have received scant attention in public health policies, including the promotion of voluntary smoke-free homes.

**Table 1:** Level of voluntary adoption of smoke-free rules at home in Barcelona (Spain) in 2013–2014 according to sociodemographics and selected smoking characteristics

		Complete indoor ban			Partial or absent indoor ban		
	n	Per cent	PR (95% CI)	PRa (95% CI)	Per cent	PR (95% CI)	PRa (95% CI)
Overall	731	57.4	–	–	42.6	–	–
*Sociodemographic variables*							
Sex							
Men	336	53.4	1	1	46.6	1	1
Women	395	60.8	1.14 (0.99 to 1.30)	1.15 (1.00 to 1.33)*	39.2	0.84 (0.70 to 1.01)	0.84 (0.71 to 1.01)
Age (years)							
26–44	299	60.4	1.09 (0.93 to 1.28)	1.11 (0.95 to 1.30)	39.6	0.88 (0.71 to 1.10)	0.89 (0.71 to 1.11)
45–64	237	55.4	1.00 (0.86 to 1.17)	1.00 (0.86 to 1.17)	44.6	1.00 (0.82 to 1.20)	1.00 (0.83 to 1.21)
65–98	195	55.2	1	1	44.8	1	1
Educational level							
Low	129	54.8	1	1	45.23	1	1
Intermediate	275	50.4	0.92 (0.76 to 1.11)	0.93 (0.76 to 1.13)	49.6	1.10 (0.82 to 1.36)	1.06 (0.83 to 1.35)
High	327	64.4	1.17 (0.99 to 1.39)	1.17 (0.97 to 1.40)	35.6	0.79 (0.62 to 1.00)	0.77 (0.58 to 1.03)
Employment							
Yes	458	61.1	1.20 (1.04 to 1.37)*	1.15 (1.00 to 1.31)	38.9	0.80 (0.67 to 0.95)*	0.69 (0.54 to 0.87)***
No	273	51.1	1	1	48.9	1	1
Married							
Yes	491	60.1	1.16 (1.00 to 1.35)	1.18 (1.01 to 1.38)*	39.9	0.83 (0.69 to 0.99)*	0.80 (0.67 to 0.97)*
No	240	51.8	1	1	48.2	1	1
Minor at home							
Yes	193	72.0	1.41 (1.23 to 1.62)***	1.40 (1.20 to 1.65)***	28.0	0.57 (0.44 to 0.75)***	0.56 (0.42 to 0.75)***
No	435	50.9	1	1	49.1	1	1
*Smoking-related variables*							
Smoking status							
Current	191	28.4	1	1	71.6	1	1
Former	250	62.7	2.21 (1.69 to 2.89)***	2.36 (1.79 to 3.10)***	37.3	0.52 (0.43 to 0.63)***	0.39 (0.34 to 0.46)***
Never	290	72.0	2.53 (1.95 to 3.29)***	2.68 (2.06 to 3.50)***	28.0	0.39 (0.32 to 0.48)***	0.31 (0.26 to 0.37)***
FTCD (among smokers)							
_ ≤_4	89	29.7	1.60 (0.65 to 3.95)	1.69 (0.68 to 4.18)	70.3	0.70 (0.60 to 0.81)***	0.78 (0.65 to 0.92)**
5	15	0.0	–	–	100.0	–	–
_ >_5	34	18.6	1	1	81.4	1	1
Intention to quit (among smokers)							
Yes	13	40.4	1.85 (0.85 to 3.99)	1.95 (0.89 to 4.28)	59.6	0.76 (0.47 to 1.23)	0.81 (0.50 to 1.31)
No	146	21.9	1	1	78.1	1	1

n not always sum up due to missing data.

*p<0.05, ** p<0.01, ***p<0.001.

PR, prevalence ratio; PRa, prevalence ratio adjusted for sex and age.

In Europe, according to the Eurobarometer 332,^8^ 61% of households had some kind of smoke-free home rules in 2009. The highest prevalence of smoke-free homes was observed in Finland (95%) and the lowest in Macedonia (30%), whereas 44% of the Spanish households had smoke-free home rules. However, these results are previous to the last smoke-free legislation in Spain (Law 42/2010),^9^ that bans smoking in public places and extends the ban to all hospitality venues without exception and to some outdoor public areas, including healthcare premises, children educational campuses and playgrounds. This new regulation makes Spain one of the countries with the most stringent national smoke-free laws in Europe.

Currently, to the best of our knowledge, there are no national descriptive studies about the adoption of smoke-free homes in Spain after the Spanish tobacco control legislations. Moreover, there is scarce evidence about the relationship between voluntary adoption of smoke-free homes and the risk perception of SHS exposure. Therefore, the objective of this study is to describe the voluntary adoption of smoke-free homes in Spain and to identify variables associated with its voluntary adoption, including risk perception towards SHS exposure.

## Methods

We used the follow-up data of a cohort study from a representative sample of the adult population (≥16 years) of the city of Barcelona (Catalonia, Spain). The objective of the cohort study was to assess the impact of the Spanish smoking bans on tobacco consumption and SHS exposure. The baseline study was carried out during the years 2004–2005 through a representative random sample of the adult (≥16 years old) non-institutionalised population of Barcelona (Spain)^10^ ^11^ (n=1245). We obtained the personal data and addresses from the updated official Census, as provided by the Institute of Statistics of Barcelona. We sent a personal letter to introduce the study; afterwards trained interviewers administered a face-to-face questionnaire (in Spanish or Catalan) at the participant's home to gather information on sociodemographic data and active and passive smoking. The follow-up took place in 2013–2014.

For this study, we exclusively used the follow-up data. From the baseline sample, we excluded 235 participants; 150 after checking their data in the Insured Central Registry of Catalonia (101 had died and 49 had migrated out of the province of Barcelona) and another 85 did not give consent to be followed up or were <18 years old in 2004–2005, because they were not legally adults at that time and we did not ask to their parents any consent to be recontacted. Follow-up was conducted between May 2013 and February 2014. In total, 72.9% of the eligible sample agreed to participate and answered the questionnaire, 18.5% (187) refused to participate, 7.2% (73) had moved elsewhere and 1.3%^14^ had died. The final sample included 736 individuals. There were statistically significant differences between the follow-up sample and the participant lost in the follow-up according to age, level of education and smoking status. Followed-up participants overestimate the young people and smokers in comparison with lost participants, for this reason, the increase in smoke-free homes could be higher among lost participants. On the other hand, the final sample overestimated the older people compared with the distribution observed in the population of Barcelona. Therefore, we used inverse probability weights to balance our data according to age distribution of the city of Barcelona to maintain its representativeness of the sample.

For this analysis, we have available data from 731 out of the 736 individuals, due to missing data in the variable of interest. The primary outcome was the voluntary adoption of smoking rules at home, which was obtained from the question: ‘Which of the following situations best describe the smoking rules in your house?’ with three possible answers: ‘Nobody can smoke’, ‘Smoking is allowed in some places’ and ‘Smoking is allowed everywhere’. According to this question, we defined smoking rules inside the households as ‘complete indoor rule’ (when smoking was not allowed inside the house), and ‘partial or absent indoor rule’ (when smoking was allowed in some designated areas inside the house or when smoking was allowed everywhere).

We also obtained information about the risk perception of SHS exposure through the degree of agreement with a set of statements: (1) SHS bothers you; (2) breathing tobacco smoke from others is harmful; (3) SHS is dangerous for adults; (4) SHS is dangerous for children; and (5) tobacco smoke is dangerous for non-smokers. The answers were collected in a five-point Likert scale (‘Totally agree’, ‘Agree’, ‘Neither agree nor disagree’, ‘Disagree’ and ‘Totally disagree’). Finally, we dichotomised each statement as ‘Agree’, indicating the participant answered either ‘Totally agree’ or ‘Agree’, and ‘Disagree’ otherwise. We also included information about nicotine dependence of smokers using the Fagerström test for cigarette dependence (FTCD).^12^

We calculated the prevalence of smoke-free rules at home, prevalence ratios (PR) and their 95% CIs, stratified by sociodemographic variables and selected smoking characteristics. We also fitted log-binomial regression models to calculate the PR, adjusted for sex and age (PRa). The statistical programs used were R-3.0.2 and STATA V.14.

## Results

About 57.4% of participants lived in households with complete indoor rules, while 42.6% lived in households with partial or absent indoor rules. Voluntary adoption of complete indoor rules at home was statistically significantly more frequent among women (PRa: 1.15; 95% CI 1.00 to 1.33), married (PRa: 1.18; 95% CI 1.01 to 1.38), never-smokers (PRa: 2.68; 95% CI 2.06 to 3.50) and in households where a minor lived (PRa: 1.40; 95% CI 1.20 to 1.65) (table 1). Similarly, voluntary adoption of partial or absent indoor rules was statistically significantly less frequent among working individuals (PRa: 0.69; 95% CI 0.54 to 0.87), married (PRa: 0.80; 95% CI 0.67 to 0.97), never-smokers (PRa: 0.31; 95% CI 0.26 to 0.37), smokers with lower FTCD score (PRa: 0.78; 95% CI 0.65 to 0.92) and participants living with a minor (PRa: 0.56; 95% CI 0.42 to 0.75) (table 1).

Smokers were those with the lowest prevalence of adoption of complete indoor smoke-free home rules. Among them, those with a medium and high dependence (FTCD), and those who did not attempt to stop smoking (table 1).

Table 2 shows the association between voluntary adoption of smoke-free homes rules and the risk perception of SHS exposure. The prevalence of complete indoor smoke-free home rules was higher among participants who perceived SHS exposure as a risk for health. Particularly, voluntary adoption of complete indoor smoke-free rules at home was statistically significantly more frequent among those who agree with the statement ‘breathing tobacco smoke from smokers is dangerous for non-smokers’ (PRa: 1.77; 95% CI 1.06 to 2.97) (table 2). Moreover, voluntary adoption of partial or absent indoor smoke-free rules at home was statistically significantly less frequent among those who agree with the statements ‘SHS bothers you’ (PRa: 0.70; 95% CI 0.50 to 0.98), ‘breathing tobacco smoke from smokers is harmful’ (PRa: 0.67; 95% CI 0.51 to 0.87), ‘SHS is dangerous for adults’ (PRa: 0.72; 95% CI 0.54 to 0.96) and ‘SHS is dangerous for non-smokers’ (PRa: 0.63; 95% CI 0.49 to 0.81) (table 2).

**Table 2:** Level of voluntary adoption of smoke-free rules at home in Barcelona (Spain) in 2013–2014, according to the perceived risk of the exposure to secondhand smoke

		Complete indoor ban			Partial or absent indoor ban		
	n	Per cent	PR (95% CI)	PRa (95% CI)	Per cent	PR (95% CI)	PRa (95% CI)
SHS bothers you							
Disagree	31	40.8	1	1	59.2	1	1
Agree	700	58.2	1.42 (0.88 to 2.30)	1.44 (0.89 to 2.33)	41.8	0.71 (0.50 to 0.99)*	0.70 (0.50 to 0.98)*
Breathing tobacco smoke from smokers is harmful							
Disagree	36	37.7	1	1	62.3	1	1
Agree	690	58.7	1.56 (1.01 to 2.40)*	1.51 (0.97 to 2.34)	41.3	0.66 (0.50 to 0.87)**	0.67 (0.51 to 0.87)**
SHS is dangerous for adults							
Disagree	35	40.7	1	1	59.3	1	1
Agree	692	58.4	1.43 (0.95 to 2.16)	1.38 (0.91 to 2.10)	41.6	0.70 (0.52 to 0.94)*	0.72 (0.54 to 0.96)*
SHS is dangerous for children							
Disagree	25	52.0	1	1	48.0	1	1
Agree	705	57.8	1.11 (0.74 to 1.67)	1.09 (0.72 to 1.66)	42.2	0.88 (0.57 to 1.37)	0.89 (0.58 to 1.38)
Secondhand smoke is dangerous for non-smokers							
Disagree	35	32.5	1	1	67.5	1	1
Agree	692	59.0	1.81 (1.09 to 3.02)*	1.77 (1.06 to 2.97)*	41.0	0.61 (0.47 to 0.79)***	0.63 (0.49 to 0.81)***

n not always sum up due to missing data.

*p<0.05, **p<0.01, ***p<0.001.

PR, prevalence ratio; PRa, prevalence ratio adjusted for sex and age.

## Discussion

More than half (57.4%) of the population of Barcelona (Spain) had complete indoor smoke-free rules at home in 2013–2014. This prevalence is higher than that obtained in the Eurobarometer^8^ (44%), maybe because the Eurobarometer considers as complete smoke-free homes those households where smoking is not allowed, without distinction between indoor and outdoor areas. On the other hand, there are some studies showing that this EU survey generates estimates that are in some cases widely discrepant from more substantive national sources and does not provide age or gender-specific data by country.^13^ Similarly, our result is also higher than what was observed in other countries like Scotland (51.8%)^3^ and the USA, where this percentage was 53% in states with lax tobacco control legislations and higher in states with comprehensive policies.^14^ Moreover, as observed in previous studies,^15–19^ the adoption of smoke-free homes in our study was higher among never-smokers and among those who lived with a minor. The prevalence of adoption of smoke-free homes among households with non-smokers was 23.5% in the UK, 39.2% in the USA, 39.1% in Canada and 44.3% in Australia.^15^ The prevalence of adoption of smoke-free home among households with infants and preprimary children was 29% and 26% in the UK, 38.9% and 51.5% in the USA, 41% and 48.8% in Canada and 60.3% and 52.7% in Australia, respectively.^15^ Our study showed that 28.4% of smokers had complete indoor smoke-free home rules. In this line, the prevalence of complete smoke-free home rules observed among smokers in other European countries is 16% in Ireland, 25% in France, 38% in Germany, 17% in the Netherlands and 25% in the UK;^19^ thus, there is room for improvement in this regard.

During the debate about the implementation of smoke-free policies in different countries, the tobacco industry and the hospitality sector argued that the restriction of smoking in public places would displace tobacco consumption to private settings, particularly to home. We have previously found a decrease in SHS exposure at home in non-smoker adults after the national comprehensive legislation.^20^ In this line, this analysis show high prevalence of complete indoor smoke-free rules (57.4%). This could be due to an increasing perception of the harmful effects of SHS exposure among the general population. In fact, we observed the highest prevalence (72%) of complete indoor smoke-free home rules in households with minors.

Our data show that the voluntary adoption of complete indoor smoke-free home rules is higher among never-smokers and among people who lived with minors. Never smokers present statistically significant higher risk perception of SHS exposure than smokers and former smokers (data not shown). This could be one reason why complete indoor smoke-free home rules are higher among never smokers. However, we found similar prevalence of SHS risk perception among people who lived with and without minors (data not shown). On the other hand, people who had some kind of risk perception of SHS exposure showed higher adoption of complete smoke-free homes rules. Similar results were obtained in a study in Italy about the support for tobacco regulation and consumption in private vehicles in the presence of minors.^21^

Our results highlight the need to increase awareness of the health risks of SHS in private settings, especially among smokers. In this regard, the awareness campaigns should inform about the health risks of SHS exposure, especially in private settings. Besides, smoking prevention among adolescents at schools should also consider including the prevention of exposure to SHS.^22^ Furthermore, it should also be reported the health benefits of having a smoke-free home by health system and social media.

The main limitation of this study is the potential of participation bias due to the attrition of the cohort of participants; our data are, particularly, older than the population of the city of Barcelona. For this reason, all analyses used weighted data to generate representative estimates of the city of Barcelona. Moreover, the study was conducted only in the city of Barcelona and generalisation of the results to the rest of Spain should be cautious. Another potential limitation is the cross-sectional nature of the data, which allow to establish associations but not to infer causality.

In conclusion, 6 out of 10 households in Barcelona (Spain) have complete indoor smoke-free rules after comprehensive tobacco control legislation in Spain. In addition, we observed an association between complete indoor smoke-free homes adoption and the perceived risk of SHS exposure. Improving the proportion of homes with smoke-free rules through different social interventions should be considered in the strategy towards the endgame.^23^ In addition, warning campaigns about the harmful effects of SHS exposure at home, especially in the presence of children, should be promoted in Spain.
